# Light-Matter Interaction in Nano Systems: Fundamentals and Applications

**DOI:** 10.3390/nano16181157

**Published:** 2026-09-15

**Authors:** Zhengkun Fu, Lianghui Huang

**Affiliations:** 1 School of Physics and Information Technology, Shaanxi Normal University, Xi’an 710062, China; 2 State Key Laboratory of Quantum Optics and Quantum Optics Devices, Institute of Opto-Electronics, School of Physics and Electronic Engineering, Shanxi University, Taiyuan 030006, China

Nanoscale light–matter interaction serves as the fundamental physical basis for modern nanophotonics, functional nanomaterials, and micro–nano optical devices, supporting a wide range of emerging applications in energy conversion, optical sensing, quantum regulation, and environmental catalysis. Benefiting from the rapid development of nanofabrication, material modification, and optical measurement technology, diverse physical phenomena including photothermal conversion, photoinduced charge transfer, luminescence modulation, and nonlinear optical response can be precisely regulated at the nanoscale [1]. Plasmonic effects, as an efficient and versatile modulation approach, can significantly enhance local light field intensity and boost light–matter coupling efficiency, enabling many novel optical and catalytic behaviors that are difficult to achieve in traditional macroscopic systems [2,3,4]. This Special Issue, entitled “Light-Matter Interaction in Nano Systems: Fundamentals and Applications”, collects eleven high-quality papers covering nanomaterial photothermal regulation, photoinduced crystal transformation, photocatalytic performance optimization, micro–nano optical field manipulation, surface-enhanced Raman sensing, and cavity-coupled quantum optical modulation. The included studies comprehensively present the latest advances in the fundamental mechanism exploration and applied technology innovation of nanoscale light–matter interaction, providing theoretical and experimental support for promoting the practical application of functional nano-optical systems.

Noble metal nanostructures and plasmonic nanocavities play a significant role in Surface-Enhanced Raman Scattering (SERS). In particular, the geometry of nanoparticle-on-mirror (NPoM) plasmonic nanocavities was investigated to understand its impact on SERS backgrounds [3]. It was discovered that laser irradiation can induce an irreversible change in the molecular conformation of the self-assembled monolayer, reducing the gap height. The resulting increased plasmon confinement not only enhances the SERS signal but also contributes to the enhancement of the background continuum via inelastic light scattering of electrons. This investigation provides new routes for modifying the geometry of plasmonic nanostructures to improve the signal-to-background ratio and SERS sensitivity. To further improve SERS sensitivity, a silver microplate–molecule–multi-sized silver nanosphere NPoM nanocavity was constructed for the high-efficiency detection of 4-mercaptobenzoic acid (4-MBA) and biphenyl-4,4′-dithiol (BPDT) molecules [4]. By precisely tuning the size of the silver nanospheres to match their resonance frequency with the Raman shift of the target molecules, the researchers achieved an efficient and stable SERS enhancement. The Raman scattering of 4-MBA and BPDT was enhanced by factors of up to 7904 and 6781, respectively, compared to individual silver nanospheres. This approach offers a valuable reference for the detection of various single-molecule layers and demonstrates significant potential for applications in biosensing and chemical analysis.

Optical nonreciprocity (ONR), which is essential for optical isolation and routing, was experimentally realized using degenerate two-level atoms embedded in an optical ring cavity [5]. By utilizing the stable ground-state Zeeman coherence for Λ-chains involved in the degenerate two-level system, the researchers demonstrated that ONR based on intracavity Electromagnetically Induced Transparency (EIT) could be achieved. The study highlighted that the cavity–atom coupling strength is weakened in the counter-propagating configuration, allowing for ONR transmission with high contrast and linewidth-narrowing at low probe intensities. This work enriches the physical application of ONR in quantum information processing.

In a related study on quantum dots, the optical properties of GaAs cone-shell quantum dots (CSQDs) under a vertical electric field were investigated [6]. By using transparent Al-doped ZnO (AZO) gates, the researchers observed a field-induced switching from a strong to an asymmetric strong–weak confinement. An increasing electric field pushes the hole into the wing part of the CSQD, forming a quantum ring where it is weakly confined, while the electron is pushed into the tip, remaining in a strong confinement. This novel asymmetric confinement was confirmed by a significant discrepancy between measured and simulated radiative lifetimes, which was resolved by modeling the asymmetric strong–weak confinement. On-chip mode routing is essential for increasing the capacity of optical networks. A silica waveguide thermo-optic mode switch with small-radius bimodal S-bends was demonstrated to implement selective E_11_ and E_21_ mode output [7]. The device, fabricated using standard CMOS processing, achieved an extinction ratio of ≥13.1 dB and a crosstalk of ≤−22.8 dB for the E_11_ mode, as well as an extinction ratio of ≥15.5 dB and a crosstalk of ≤−18.1 dB for the E_21_ mode. This work proves the feasibility of using multimode S-bends in mode switching and holds promise for on-chip mode-division multiplexing (MDM) applications.

In the field of photovoltaics, interfacial engineering of the electron transport layer (ETL) is critical for device performance. An investigation into SnO_2_-based interfacial engineering towards improved perovskite solar cells (PSCs) was conducted by adjusting the concentration of aqueous SnO_2_ [8]. It was found that the concentration of SnO_2_ significantly affects the surface morphology, conductivity, and trap density of the perovskite layer. The device prepared with an optimized SnO_2_ concentration of 2.4% showed the highest average Power Conversion Efficiency (PCE) of 20.27%. This work provides a comprehensive understanding of how SnO_2_ concentrations influence crystallization kinetics and charge transport in PSCs.

The development of novel light sources and their control mechanisms is another key theme. A new type of nano-source of light, the quantum cone, was proposed, which is composed of multiple quantum dots with gradually decreasing diameters [9]. This structure produces a dispersive radiated spectrum, with the blue edge determined by quantum confinement at the cone’s top and the red edge by the semiconductor’s bandgap. The quantum cone acts as an ultrafast nano-monochromator, substituting for both a light source and a dispersive element in a conventional spectrometer, enabling the construction of nano-spectrometers for measuring the absorbance spectra of virus and molecule particles.

The unique optical properties of nanomaterials also extend to biomedical applications. The effects of immersing Cr^3+^-doped ZnGa_2_O_4_ (CZGO) nanoparticles, a promising material for near-infrared persistent luminescence (PersL) bioimaging, in a simulated physiological medium were investigated [10]. Interestingly, the luminescence intensity and lifetime of the CZGO nanoparticles noticeably improved after immersion in a phosphate-buffered saline (PBS) solution for up to 48 h. This enhancement was attributed to the formation and crystallization of a ZnO layer on the nanoparticle surface, which passivates surface defects. This finding suggests that CZGO nanoparticles are a robust and promising candidate for bioimaging applications within physiological environments.

The collection is further enriched by studies on advanced material growth and nonlinear photonics. High-quality GaP(111) thin films were successfully grown using gas-source molecular-beam epitaxy (GSMBE), achieving atomically smooth surfaces essential for photonic applications [11]. This work demonstrates the fabrication of photonic crystal cavities from GaP(111) with a quality factor (Q) of 1200. Given GaP’s high refractive index, wide optical bandgap, and pronounced optical anisotropy, these findings highlight its significant potential for advanced photonic architectures, particularly in applications requiring strong light confinement and nonlinear optical processes like second-harmonic generation.

Overall, this collection provides a selected group of papers that covers various aspects of nanophotonics, ranging from plasmonic sensing and quantum optics to photovoltaic interfaces and laser manufacturing; we sincerely hope that the reader will benefit from such an informative and insightful compilation.
